# Real‐World Experience Using Bimekizumab in a Patient Cohort With Plaque‐Type Psoriasis and Chronic Kidney Disease: A 48‐Week Retrospective Multicentre Study

**DOI:** 10.1111/ijd.17657

**Published:** 2025-01-13

**Authors:** Zeno Fratton, Anna Balato, Stefano Bighetti, Luca Bettolini, Vincenzo Maione, Piergiacomo Calzavara‐Pinton, Dario Buononato, Giuseppe Stinco, Enzo Errichetti

**Affiliations:** ^1^ Institute of Dermatology, Department of Medicine University of Udine Udine Italy; ^2^ Dermatology Unit University of Campania Luigi Vanvitelli Naples Italy; ^3^ Dermatology Department University of Brescia, ASST Spedali Civili di Brescia Brescia Italy

**Keywords:** bimekizumab, chronic kidney disease, efficacy, psoriasis, safety

Psoriasis is associated with an increased risk of chronic kidney disease (CKD), with a prevalence of 1%–8% [1]. While biologics generally do not alter renal function [2], no data on bimekizumab, an interleukin (IL)‐17A/F inhibitor, are currently available for patients with CKD.

Our 48‐week retrospective study across three Italian dermatological centers (Udine, Brescia, and Naples) assessed the efficacy and safety of bimekizumab in patients with moderate‐to‐severe psoriasis and CKD, alongside its impact on renal function. Consecutive psoriatic patients with a history of CKD treated with bimekizumab (standard dosing regimen) for more than 4 weeks were considered; we excluded patients undergoing concomitant anti‐psoriatic therapies (systemic or topical). Data on demographics, medical history, and renal function (serum creatinine estimated Glomerular Filtration Rate (eGFR) according to the CKD‐EPI formula) were collected. The Psoriasis Area Severity Index (PASI) was evaluated at baseline and Week 4, as well as Weeks 16, 24, and 48 when available. The safety profile was also evaluated. A paired *t*‐test was used to assess significant differences in serum creatinine variations. The normality of data was checked through the Shapiro–Wilk test. All analyses were performed using R Statistical Software (v4.1.2; R Core Team 2021). This study was conducted in accordance with the 1975 Declaration of Helsinki's ethical standards.

Fifteen patients with a history of CKD were included in our study; 10 were male (66.7%). The mean age of the patients was 64.3 ± 12.8 years. At baseline, the mean PASI was 18.8 ± 11.4, and 10 patients (66.7%) had one or more difficult‐to‐treat areas (genitalia, scalp, palms/soles). Six patients (40.0%) had nail involvement. Fourteen (93.3%) patients had received prior conventional therapy. Twelve patients (80.0%) had previously received at least one biologic, and five (33.3%) had previously failed at least three biologics (mean biologic failure of 1.9 ± 1.6 per patient).

CKD stages ranged from two (six patients) to five (one patient treated with hemodialysis). Eight (53.3%) and five (33.3%) patients had arterial hypertension and diabetes mellitus, respectively. Detailed baseline demographic and clinical characteristics of the included patients are summarized in Table 1.

**TABLE 1:** Summary of demographic and clinical characteristics of included patients.

Patients, *n*	15
Age, years	64.3 (±12.8)
Sex, male	10 (66.7%)
Body Mass Index, kg/m^2^	28.6 (±3.6)
Psoriasis duration, years	12.5 (10.3)
Psoriasis family history, *n* (%)	7 (46.7%)
Difficult‐to‐treat areas, *n* (%)	10 (66.7%)
Scalp/genitals/palms and soles/nails	6 (40%)/4 (26.7%)/1 (6.7%)/6 (40%)
Baseline PASI	18.6 (±11.4)
CKD stage,^a^ *n* (%)	
Stage 2	6 (40%)
Stage 3a	4 (26.7%)
Stage 3b	4 (26.7%)
Stage 4	0 (0.0%)
Stage 5	1 (6.7%)
Serum Creatinine ± SD (eGFR)^b^	
Baseline	1.47 ± 0.4 mg/mL (53.2 mil/min per 1.73 m^2^)
Week 24	1.44 ± 0.4 mg/mL (54.6 mil/min per 1.73 m^2^)
Other comorbidities, *n* (%)	
Hypertension	8 (53.3%)
Diabetes	5 (33.3%)
Biologics failure, *n* (%)	
Naive	3 (20.0%)
1 failure	5 (33.3%)
2 failures	2 (13.3%)
3+ failures	5 (33.3%)
Failures per patient, *n* ± SD	1.9 (±1.6)
*Adverse events, n (%); week of onset*	*Total: 6 (40%)*
Candidiasis	2 (13.3%); W8
Pneumonia with evolution to sepsis	1 (6.7%); W41
Urinary sepsis	1 (6.7%); W13
Urticaria (discontinuation at Week 8)	1 (6.7%); W4
Fever with aphthae and vasculitis	1 (6.7%); W20

*Note:* CKD, chronic kidney disease; eGFR, estimated Glomerular Filtration Rate; PASI, Psoriasis Area Severity Index, SD, standard deviation; W, week.

^b^ Calculated with CKD‐EPI formula.

During the follow‐up (Figure 1), mean PASI decreased from 18.8 ± 11.14 at baseline to 5.7 ± 4.7 (Week 4), 2.8 ± 4.0 (Week 16), 1.6 ± 1.8 (Week 24) and 0.2 ± 0.6 (Week 8). In terms of the improvement percentage, PASI75, PASI90, and PASI100 were achieved by 53.3%, 13.3%, and 13.3% at Week 4; 66.7%, 66.7%, and 55.6% at Week 16; 100%, 69.2%, and 46% at Week 24, and 100%, 90.9%, and 90.9% at Week 48, respectively.

**FIGURE 1:**
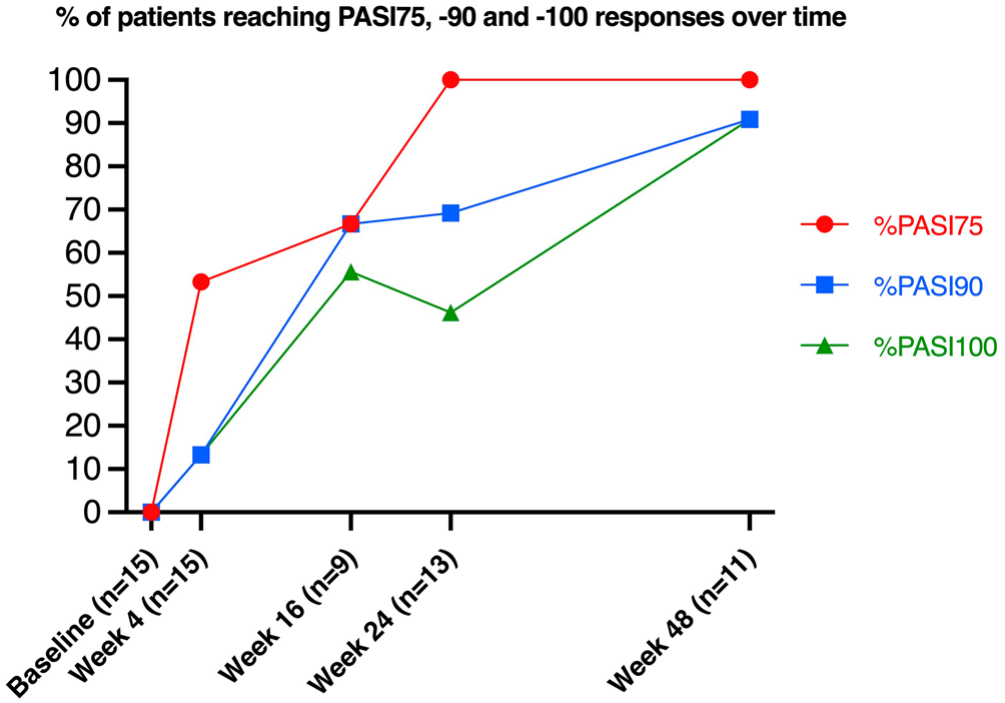
Psoriasis Area Severity Index (PASI) responses at follow‐up visits. PASI 75, 90, and 100 responses after 4, 16, 24, and 48 weeks of bimekizumab treatment. Follow‐up at Week 16 was available for nine patients. Follow‐up for one patient was available only until Week 4. One patient discontinued bimekizumab at Week 8.

Serum creatinine levels did not vary significantly (*p* value = 0.3977) during the treatment period (mean serum creatinine 1.47 ± 0.4 mg/mL at baseline, 1.44 ± 0.4 mg/mL at Week 24).

Six patients (40.0%) experienced adverse events (AEs). Treatment discontinuation occurred in one patient due to urticaria. Two patients developed mild candidiasis, which was successfully treated topically. Two patients (one with a history of recurrent urinary infections) were hospitalized due to urinary sepsis and pneumonia with subsequent sepsis and successfully treated with appropriate management without altering the treatment administration schedule. One patient developed fever, aphthous ulcers, and vasculitis, which were successfully managed with systemic treatment. Most AEs (4/6, 66.7%) occurred in the first 16 weeks of treatment.

In conclusion, this study suggests that bimekizumab effectively treats moderate‐to‐severe psoriasis in patients with CKD. While outcomes at Week 48 were comparable to other real‐world studies [3], early PASI responses (Week 4 and 16) were slower [4], suggesting a possible impact of CKD on treatment outcomes, a finding that had not been documented before.

Finally, AEs were frequent, consistent with what has already been described in patients with renal impairment [5]. While candidiasis has been extensively described as one of the common AEs associated with bimekizumab treatment, the association with the other AEs described in our study is not as strong. Future studies with larger cohorts and longer follow‐up periods are needed to validate our findings.

## Consent

Informed consent was obtained from all patients involved in the study.

## Conflicts of Interest

Anna Balato has served as a consultant and has received fees from Abbvie, Almirall, Amgen, BMS, Boehringer Ingelheim, Eli‐Lilly, Leo‐Pharma, Novartis, Sanofi, and UCB. The other authors have no conflicts of interest to declare.
